# Supplementary material for: Bullying prevalence in Pakistan’s educational institutes: Preclusion to the framework for a teacher-led antibullying intervention
Source: PLoS One. 2023 Apr 27;18(4):e0284864. doi: 10.1371/journal.pone.0284864 (PMC10138218; doi:10.1371/journal.pone.0284864)
Supplement: Attachment — Submitted filename: Review Report 1.docx [file pone.0284864.s001.docx]

**Review Report**

PLOS One Manuscript No. PONE-D-22-34325

The research paper entitled “Bullying prevalence in Pakistan’s educational institutes and recommendations for tailored interventions: Preclusion to the framework for a teacher-led antibullying intervention” submitted to PLOS one was reviewed. Although it is an interesting study, yet it has some flaws which are necessary to be removed to improve the quality of this paper. These flaws are briefly stated as under: -

1. While citing (quoting) different studies in this paper, the author used Nos which disturb the flow of text and confuse the readers. For example, the author’s style of citation like according to 60 and 70 is appeared inadequate. Instead, the name of the authors and years of publication are more suitable. It will save the time of reader to see every number in references. The number can be given at the end of quoted study.

2. Independent and dependent variables are not clearly defined for the understanding of readers.

3. The explanation of Table 7 ANOVA results and Table 8 Multiple comparisons are very short.

The results of these tables may be illustrated properly.

4. Recommendations and suggestions may be given after conclusions.

5. The conclusions are very brief and confusing. The author should elaborate what conclusion

he/she has drawn from his findings.

6. Anti-bullying techniques may be described one be one in bullets so that the academicians can

study and apply them in their respective educational institutions.

7. The author must write practical implications of this study, which are missing.

8. There is a lack of sequency in the text and grammatical mistakes which may be removed..

9. Theoretical foundation of this study is weak. It may be strengthened by adding some latest

theories in the study.

**Recommendations:** Minor revisions are required.
