# Supplementary material for: Bullying prevalence in Pakistan’s educational institutes: Preclusion to the framework for a teacher-led antibullying intervention
Source: PLoS One. 2023 Apr 27;18(4):e0284864. doi: 10.1371/journal.pone.0284864 (PMC10138218; doi:10.1371/journal.pone.0284864)
Supplement: Attachment — Submitted filename: Response to Reviewers Comments.docx [file pone.0284864.s002.docx]

**Response to Reviewers’ Comments**

**PLOS One Manuscript No. PONE-D-22-34325**

| **Reviewers’ Comments** | **Response** |
| --- | --- |
| **Reviewer 1** |  |
| Title of the research study is too lengthy, therefore, it needs to be shortened. | Title is shortened as:  Bullying prevalence in Pakistan’s educational institutes: Preclusion to the framework for a teacher-led antibullying intervention |
| The citations should be properly done. For instance, [17] enumerated that the level of cyberbullying has substantially increased in educational 95 institutions in Pakistan. Before [17] the name of the author should be properly written in the correct style. | Corrections have been made throughout the manuscript. |
| To enrich the literature Review, cite the following relevant studies <https://papers.ssrn.com/sol3/papers.cfm?abstract_id=2599095> <https://papers.ssrn.com/sol3/papers.cfm?abstract_id=2600403> <https://papers.ssrn.com/sol3/papers.cfm?abstract_id=2602997> <https://papers.ssrn.com/sol3/papers.cfm?abstract_id=2602965> <https://papers.ssrn.com/sol3/papers.cfm?abstract_id=2602568> | First two and fourth articles are cited and referred. Third and fifth articles are about children with special needs which is beyond the scope of this study. |
| Recommendations and Limitations should be written separately under the conclusion. | Limitations and recommendations for future research are added after conclusion. |
| **Reviewer 2** |  |
| The author must remove the pin pointing flaws in the paper in order to improve its quality. These flaws are minor in nature but are very important to improve the quality of paper. | Corrections have been made as suggested. Grammatical errors are also removed. |
| **Review Report 1** |  |
| While citing (quoting) different studies in this paper, the author used Nos which disturb the  flow of text and confuse the readers. For example, the author’s style of citation like according to  60 and 70 is appeared inadequate. Instead, the name of the authors and years of publication are  more suitable. It will save the time of reader to see every number in references. The number can  be given at the end of quoted study. | Corrections have been made throughout the manuscript |
| Independent and dependent variables are not clearly defined for the understanding of readers. | Several different types of statistical tests are conducted and reported in this study, and in each test, dependent and independent variables are not required. For example, tables 3, 4, 5 and 9 are purely descriptive in nature, so independent and dependent variables are not mentioned. In Table 6, the Pearson Correlation test shows the association or relationship between two variables (the terms dependent and independent may be confusing here). Furthermore, we assume that the relationship is linear, that one variable increases or decreases by a fixed amount for every unit increase or decrease in the other. Dependent and independent variables are listed in the tables for the ANOVA test (tables 7 and 8). Further information in the text is also added. |
| The explanation of Table 7 ANOVA results and Table 8 Multiple comparisons are very short.  The results of these tables may be illustrated properly. | Additions have been made as suggested. |
| Recommendations and suggestions may be given after conclusions. | Limitations and recommendations for future research are added after conclusion. |
| The conclusions are very brief and confusing. The author should elaborate what conclusion  he/she has drawn from his findings. | Some more explanations and a summary of the findings have been added. |
| Anti-bullying techniques may be described one be one in bullets so that the academicians can  study and apply them in their respective educational institutions. | Dear Reviewer, I appreciate your comment, but this paper is designed to highlight the results of the survey that was conducted before the planning and implementation of teachers-led antibullying interventions. Some of the recommendations for the upcoming intervention are already discussed under the heading Recommendations and suggestions in numbered form (not in bullet). Intervention techniques developed after baseline need assessment will be discussed in the next research paper which is currently under publication in one of the other research journals. |
| The author must write practical implications of this study, which are missing. | Practical implications have been added before conclusion. |
| There is a lack of sequency in the text and grammatical mistakes which may be removed.. | In order to remove errors, thorough proofreading has been performed and a thorough language editing has been conducted. |
| Theoretical foundation of this study is weak. It may be strengthened by adding some latest  theories in the study. | Thank you for your suggestion. Some of the theories associated with the behaviour of bullying and aggression are added to the literature review section "**The theoretical basis for explaining the concept of bullying and aggression"** |
