# Supplementary material for: Bullying prevalence in Pakistan’s educational institutes: Preclusion to the framework for a teacher-led antibullying intervention
Source: PLoS One. 2023 Apr 27;18(4):e0284864. doi: 10.1371/journal.pone.0284864 (PMC10138218; doi:10.1371/journal.pone.0284864)
Supplement: Attachment — Submitted filename: Response to Reviewers Comments.docx [file pone.0284864.s003.docx]

**Response to Reviewers’ Comments**

**PLOS One Manuscript No. PONE-D-22-34325**

| **Reviewers’ Comments** | **Response** |
| --- | --- |
| **Revisions 1** |  |
| **Reviewer 1** |  |
| Title of the research study is too lengthy, therefore, it needs to be shortened. | Title is shortened as:  Bullying prevalence in Pakistan’s educational institutes: Preclusion to the framework for a teacher-led antibullying intervention |
| The citations should be properly done. For instance, [17] enumerated that the level of cyberbullying has substantially increased in educational 95 institutions in Pakistan. Before [17] the name of the author should be properly written in the correct style. | Corrections have been made throughout the manuscript. |
| To enrich the literature Review, cite the following relevant studies <https://papers.ssrn.com/sol3/papers.cfm?abstract_id=2599095> <https://papers.ssrn.com/sol3/papers.cfm?abstract_id=2600403> <https://papers.ssrn.com/sol3/papers.cfm?abstract_id=2602997> <https://papers.ssrn.com/sol3/papers.cfm?abstract_id=2602965> <https://papers.ssrn.com/sol3/papers.cfm?abstract_id=2602568> | First two and fourth articles are cited and referred. Third and fifth articles are about children with special needs which is beyond the scope of this study. |
| Recommendations and Limitations should be written separately under the conclusion. | Limitations and recommendations for future research are added after conclusion. |
| **Reviewer 2** |  |
| The author must remove the pin pointing flaws in the paper in order to improve its quality. These flaws are minor in nature but are very important to improve the quality of paper. | Corrections have been made as suggested. Grammatical errors are also removed. |
| **Review Report 1** |  |
| While citing (quoting) different studies in this paper, the author used Nos which disturb the  flow of text and confuse the readers. For example, the author’s style of citation like according to  60 and 70 is appeared inadequate. Instead, the name of the authors and years of publication are  more suitable. It will save the time of reader to see every number in references. The number can  be given at the end of quoted study. | Corrections have been made throughout the manuscript |
| Independent and dependent variables are not clearly defined for the understanding of readers. | Several different types of statistical tests are conducted and reported in this study, and in each test, dependent and independent variables are not required. For example, tables 3, 4, 5 and 9 are purely descriptive in nature, so independent and dependent variables are not mentioned. In Table 6, the Pearson Correlation test shows the association or relationship between two variables (the terms dependent and independent may be confusing here). Furthermore, we assume that the relationship is linear, that one variable increases or decreases by a fixed amount for every unit increase or decrease in the other. Dependent and independent variables are listed in the tables for the ANOVA test (tables 7 and 8). Further information in the text is also added. |
| The explanation of Table 7 ANOVA results and Table 8 Multiple comparisons are very short.  The results of these tables may be illustrated properly. | Additions have been made as suggested. |
| Recommendations and suggestions may be given after conclusions. | Limitations and recommendations for future research are added after conclusion. |
| The conclusions are very brief and confusing. The author should elaborate what conclusion  he/she has drawn from his findings. | Some more explanations and a summary of the findings have been added. |
| Anti-bullying techniques may be described one be one in bullets so that the academicians can  study and apply them in their respective educational institutions. | Dear Reviewer, I appreciate your comment, but this paper is designed to highlight the results of the survey that was conducted before the planning and implementation of teachers-led antibullying interventions. Some of the recommendations for the upcoming intervention are already discussed under the heading Recommendations and suggestions in numbered form (not in bullet). Intervention techniques developed after baseline need assessment will be discussed in the next research paper which is currently under publication in one of the other research journals. |
| The author must write practical implications of this study, which are missing. | Practical implications have been added before conclusion. |
| There is a lack of sequency in the text and grammatical mistakes which may be removed.. | In order to remove errors, thorough proofreading has been performed and a thorough language editing has been conducted. |
| Theoretical foundation of this study is weak. It may be strengthened by adding some latest  theories in the study. | Thank you for your suggestion. Some of the theories associated with the behaviour of bullying and aggression are added to the literature review section "**The theoretical basis for explaining the concept of bullying and aggression"** |
| **Revisions 2** |  |
| **Journals Requirements:**  Please review your reference list to ensure that it is complete and correct. If you have cited papers that have been retracted, please include the rationale for doing so in the manuscript text, or remove these references and replace them with relevant current references. Any changes to the reference list should be mentioned in the rebuttal letter that accompanies your revised manuscript. If you need to cite a retracted article, indicate the article’s retracted status in the References list and also include a citation and full reference for the retraction notice. | Reference list is checked once again and is correct.  Some of the papers were cited as per reviewer’s suggestion. The references for those papers have been updated and checked as well. The new references are mainly added in theoretical framework in literature review and some references were suggested by Reviewer 1 which are also added and referred.  Reference list is updated. |
| **Revisions 3** |  |
| ACADEMIC EDITOR:  Please do the suggested changes  For Lab, Study and Registered Report Protocols: These article types are not expected to include results but may include pilot data. | The manuscript is not a lab study or a registered report protocol. The article is based on a survey conducted in Pakistan from teachers about their opinions and experiences of bullying in education. In this study validated and reliable questionnaires were used so piloting was not carried out before data collection and results are reported after full data collection completed. |
| **I did not find any revisions in email or on portal. I assume that only I have to check references in the revised version of manuscript and address academic editor comments. The reviews received after the first decision are already addressed in first revised version. However, it has been uploaded again in this submission.** | |
